# Insights into Photo Degradation and Stabilization Strategies of Antibody–Drug Conjugates with Camptothecin Payloads

**DOI:** 10.3390/pharmaceutics17111397

**Published:** 2025-10-28

**Authors:** Shukun Luo, Joshua Bulos, Ricky Uroza, Yimeng Zhao, Xiao Pan, Yue Su, Haibo Qiu, Babatunde Olagunju, Wenhua Wang, Dingjiang Liu, Mohammed Shameem

**Affiliations:** 1 Formulation Development, Regeneron Pharmaceuticals, Inc., 777 Old Saw Mill River Road, Tarrytown, NY 10591, USA; shukun.luo@regeneron.com (S.L.); joshua.bulos@regeneron.com (J.B.); ricky.uroza@regeneron.com (R.U.); mohammed.shameem@regeneron.com (M.S.); 2 Analytical Chemistry Group, Regeneron Pharmaceuticals, Inc., 777 Old Saw Mill River Road, Tarrytown, NY 10591, USA; yimeng.zhao@regeneron.com (Y.Z.); xiao.pan@regeneron.com (X.P.); yue.su@regeneron.com (Y.S.); haibo.qiu@regeneron.com (H.Q.); 3 Department of Chemistry, State University of New York College of Environmental Science and Forestry, Syracuse, NY 13210, USA

**Keywords:** photostability, antibody–drug conjugates (ADCs), camptothecin (CPT), deruxtecan (DXd), payload degradation, histidine degradation, antioxidant, methionine, ascorbic acid

## Abstract

**Background**: Photostability assessment is a critical component in the development of drug products, particularly for antibody–drug conjugates (ADCs) containing light-sensitive small molecules such as camptothecin (CPT) and its derivatives. ADCs conjugated with CPT derivative payloads often require extensive formulation and drug product development to ensure product stability due to their unique light-induced degradation pathways. In this study, we assessed the photostability of two ADC molecules with a CPT derivative payload (deruxtecan, DXd). **Methods**: Following light exposure, the stability of ADCs was assessed by examining critical quality attributes, such as aggregation and photodegradation products of the antibody, payload, and formulation excipients, using advanced liquid chromatography and mass spectrometry techniques. **Results**: Our results revealed key degradation pathways, including the formation of high-molecular-weight (HMW) species, payload degradation, and post-translational modifications (PTMs) on amino acid residues in the antibodies. Additionally, the DXd payload amplified the photosensitivity of the formulation solution, leading to histidine degradation in the formulation buffer and subsequent pH changes. To enhance the stability of ADCs for manufacturing and therapeutic use, we developed a robust formulation by systematic buffer screening and a targeted evaluation of selected antioxidant excipients. Further investigations into light conditions revealed that DXd ADCs are particularly sensitive to short-wavelength light. When evaluating the container closure system, it was demonstrated that using amber vials is a viable option for protecting against light-induced degradation. **Conclusions**: This report outlines a comprehensive strategy to address photo instability in DXd ADC drug product development, focusing on formulation optimization, controlled manufacturing light settings, and the option of using protective containers to ensure product stability.

## 1. Introduction

An antibody–drug conjugate (ADC) is a type of highly effective therapeutic modality designed to selectively target cancer cells while minimizing systemic toxicity to healthy tissues. ADCs consist of three key components: an antibody or antibody fragment that binds specifically to tumor-associated antigens, a cytotoxic payload that induces cell death, and a chemical linker that connects the two. This design allows for the targeted delivery of potent cytotoxic agents, thereby reducing off-target toxicity compared with conventional chemotherapy. Among the many cytotoxic payloads used in ADCs, camptothecin (CPT) has shown particular promise due to its ability to inhibit topoisomerase I, a critical enzyme in DNA replication [1]. CPT-based ADCs not only exhibit direct cytotoxic effects on cancer cells with binding targets but also induce cell death of the neighboring cancer cell through the bystander effect, making them attractive for clinical development [2].

Photostability of pharmaceutical products is usually assessed based on the ICH guidance (ICH Q1B guidance, Photostability Testing of New Drug Substances and Products). For therapeutic proteins, photo degradation can manifest through various degradation pathways, including high-molecular-weight (HMW) aggregate formation [3,4], degradation of buffer components [5,6], and post-translational modifications (PTMs) on the antibodies [7,8]. These light-induced degradation pathways can cause long-term stability issues in drug products [9]. Studies have shown that aggregate formation can reduce therapeutic efficacy and potentially increase immunogenicity [10,11]. Furthermore, light-induced oxidative stress can trigger PTMs on the protein, such as methionine and tryptophan oxidation [12], which may impact antibody structure [4] and the antigen-binding affinity if the PTMs occur in the Complementarity-Determining Regions (CDRs). PTMs on the Fc region, especially oxidation on the methionine residues located on the CH2-CH3 interface, were reported to affect the binding of antibodies to Fcγ receptors (FcγR) and to the neonatal Fc receptor (FcRn), leading to impaired effector function and serum half-life [13,14,15,16]. Degradation of buffer components, such as tris or histidine [5,6], can further exacerbate these degradation processes by promoting the formation of adducts and influencing the pH of buffers.

Camptothecin (CPT) and its derivatives exhibit inherent fluorescent features due to their chemical structure, leading to photo instability [17,18]. When exposed to light, CPT and its derivatives can undergo photo degradation, resulting in the formation of inactive degradation products [18]. These degradations can compromise the therapeutic efficacy of ADCs and pose safety risks. Moreover, photo instability poses significant challenges to the development of stable and effective formulations for ADCs using CPT and its derivatives as payloads. In general, CPT and its derivatives act as photosensitizers, generating reactive species upon light exposure [17]. These reactive species can potentially damage the antibody, the linker payload, or buffer components, and thereby accelerate degradation and destabilize the entire ADC molecule. In addition, degradation of the linker payload may result in premature release of the payload, leading to off-target toxicity or reduced drug delivery to the tumor cells [19,20]. As a result, addressing photostability in CPT-based ADCs is essential for ensuring their stability and efficacy.

In this study, we have assessed the stability of two ADC molecules with a CPT derivative payload (deruxtecan, DXd). Our results confirmed that light exposure triggers several degradation pathways of the ADCs with DXd payload (hereafter, DXd ADCs). To improve photostability, we have screened several formulation buffers, including histidine, phosphate, MES, citrate, and succinate. Our data indicates that histidine provides the best protection from light as an oxidative scavenger. We have also evaluated several excipients, including sucrose, methionine, and ascorbic acid, and revealed that these excipients are beneficial for photostability of DXd ADCs. In addition, the photostability of DXd ADCs was characterized under multiple light settings, including cool white (CW) light, UV-A, light-emitting diode (LED) light (4000K), and light with specific wavelengths generated by bandpass filters. Our results showed that DXd ADCs are more sensitive to UV-A and blue light compared to other wavelengths, providing insights into safe light settings for manufacturing and clinical administration. Finally, we compared several drug substance and drug product containers, including two types of polycarbonate (PC) bottles, clear glass vials, multi-layer cyclic olefin polymer (COP) vials with reduced UV transmittance, and amber glass vials. Our results showed that the amber and COP glass vials provide better light protection than clear glass vials, while no protection differences were observed between the two types of PC bottles. Together, our study reveals the major degradation pathways under light exposure and provides mitigation strategies to improve the photostability of DXd ADCs for drug product development and manufacturing.

## 2. Materials and Methods

### 2.1. Materials

The antibodies (IgG4) were produced in Chinese hamster ovary cells using standard manufacturing processes at Regeneron Pharmaceuticals, Inc. (Tarrytown, NY, USA). The DXd ADCs were produced by conjugating deruxtecan (DXd) to the antibodies using a cleavable linker in a site-specific manner at Regeneron Pharmaceuticals, Inc. All chemicals and solvents used for formulation preparation and sample analysis were ACS-grade. Histidine (catalog # 32924) and histidine hydrochloride monohydrate (catalog # 32930) were obtained from Ajinomoto (Tokyo, Japan). Sucrose (catalog # S-124-2-MC) and methionine (catalog # M-168) were obtained from Ferro Pfanstiehl (Waukegan, IL, USA), and MES acid (catalog # 475893-1KG) and MES sodium salt (catalog # 475894-1KG) from EMD Millipore (Burlington, MA, USA). Succinic acid (catalog # 1.00681.5000) and disodium succinate (catalog # 8.2015.0100) were purchased from Sigma-Aldrich (St. Louis, MO, USA). Ascorbic acid (catalog #0938-05), citric acid (catalog # 0115-01), trisodium citrate (catalog # 3649-01), tris hydrochloride (catalog # 4106-01), tris base (catalog # 4109-01), sodium chloride (catalog # 3628-01), sodium phosphate monobasic (catalog # 3820-01), sodium phosphate dibasic (catalog #3817-01) and trifluoroacetic acid (catalog # 9470-00) were purchased from J.T. Baker (Phillipsburg, NJ, USA). Acetonitrile (catalog # 733466) was purchased from Fisher Scientific (Waltham, MA, USA). Urea (catalog # U0631) was purchased from Millipore Sigma (Billerica, MA, USA). Sodium perchlorate (catalog # BT138240-500G) was purchased from Bean Town Chemical (Hudson, NH, USA). The water used in making buffers was purified using a Millipore Milli-Q system (Millipore SAS, Molsheim, France). Nalgene and Cellon PC bottles were purchased from ThermoFisher (Waltham, MA, USA) and Sanisure (Camarillo, CA, USA), respectively. Both clear and amber glass vials were purchased from Schott (Rye Brook, NY, USA), and COP vials were purchased from Mitsubishi Gas Chemical (New York, NY, USA). The papain enzyme (catalog # 07465) was purchased from Stemcell Technologies (West Valley, WA, USA). Sequencing-grade modified trypsin enzyme (catalog # V5111) was purchased from Promega (Madison, WI, USA).

### 2.2. Light Exposure Studies

Formulated materials (0.4 mL) were filled into 2 mL clear glass vials for all the studies, except the container studies, where they were filled with 1 mL of material. The materials were then exposed to either CW light with a variety of doses from 48 to 600 klux*hours or UV-A light in the dose ranges of 0 to 43.2 W·h/m^2^ at 20 °C using a Bahnson (ES2000) light chamber (Bahnson Environmental Specialties, Raleigh, NC, USA). Control samples were wrapped with aluminum foil alongside the light-exposed samples. The LED light exposure studies were performed in self-assembled equipment with temperature control.

### 2.3. Light Wavelength Study

Bandpass filters were used to generate light within specific wavelength ranges. Bandpass filters were purchased from Alluxa (Santa Rosa, CA, USA) with catalog numbers 7377, 7102, 7175, 7127, and 7147. Samples were placed in a 3D-printed apparatus wrapped in aluminum foil, with the top covered by different bandpass filters, and then stored in the light chamber with 144 klux*hours CW light.

### 2.4. Size-Exclusion Ultra-Performance Liquid Chromatography (SE-UPLC)

A Waters Acquity (Milford, MA, USA) system with a Waters Acquity UPLC BEH200 SEC column was used for SE-UPLC analysis. A typical experiment was set at a flow rate of 0.3 mL/min, with a mobile phase containing 10 mM phosphate, pH 6.0, and 1 M sodium perchlorate. The UV absorbance at 280 nm was monitored for %HMW quantitation and the absorption at both 280 and 360 nm was used to obtain the average drug-to-antibody ratio (DAR).

### 2.5. Papain Digestion Reaction

The ADC B samples were diluted from a concentration of 25 mg/mL to 1 mg/mL using a buffer solution containing 10 mM histidine at pH 5.2. To initiate the enzyme digestion reaction, 50 µL of papain (0.5 mg/mL) was added to 950 µL of the diluted ADC B solution. The mixture was then incubated at 37 °C for 10 min.

Free drug-related impurity (FDRI) analysis by reversed-phase ultra-performance liquid chromatography (RP-UPLC) and degradation product identification by liquid chromatography coupled with mass spectrometry (LC-MS).

The proteins in the papain digestion system were precipitated by adding 120 uL of acetonitrile (ACN) to 50 uL of the sample. The mixture was incubated at room temperature for 10 min to allow precipitation, followed by centrifugation at ≥15,000× *g* for 10 min. The supernatant was then transferred to a vial for FDRI analysis by RP-UPLC. RP-UPLC analysis was performed on a Waters Acquity (Milford, MA, USA) system with a Waters Acquity BEH C18 column. The experiment was conducted at a flow rate of 0.4 mL/min, with mobile phase A containing 0.05% trifluoroacetic acid (TFA) in water and mobile phase B containing 0.05% TFA in ACN. The UV absorbance at 360 nm was monitored for quantitation. LC-MS was used to further characterize the cleaved free drug and its degradants using the same FDRI method coupled to a Q Exactive^TM^ Plus Hybrid Quadrupole-Orbitrap ^TM^ Mass Spectrometer (Thermo, Waltham, MA, USA). The cleaved small molecule and its degradants were identified by their accurate masses.

### 2.6. Peptide Mapping by LC-MS/MS

LC-MS/MS peptide mapping was employed to identify PTMs and linker payload degradation. Naked antibody and ADC samples were denatured and reduced in 5 mM acetic acid and 5 mM tris (2-carboxyethyl) phosphine (TCEP) by heating at 80 °C for 10 min. The reduced samples were alkylated with 1 mM indole-3-acetic acid (IAA) and digested with trypsin at an enzyme-to-substrate ratio of 1:20 (*w*/*w*) for 3 h at 37 °C followed by quenching with TFA. Online LC-MS/MS analyses were performed on a Waters ACQUITY UPLC I-Class system (Milford, MA, USA). Peptides separated by the column were detected by UV absorption at 215 nm and 360 nm and subjected to MS acquisition using a Q Exactive^TM^ Plus Hybrid Quadrupole-Orbitrap^TM^ Mass Spectrometer (Thermo, Waltham, MA, USA). Peptide identification was performed by Byos software from Protein Metrics (version 5.4.52, Cupertino, CA, USA) based on accurate masses and MS/MS fragmentation of peptides. Carbamidomethylation of cysteine was included as a fixed modification in the search parameters while common antibody PTMs were included as variable modifications. Peptide quantification was performed using Skyline software (version 23.1.1.520, MacCoss Lab Software, Seattle, WA, USA).

### 2.7. Histidine Analysis LC-MS

LC-MS was used in histidine buffer degradation analysis. Antibody or ADC samples were diluted with water and injected into a Waters ACQUITY UPLC I-Class system with an HS F5 HPLC column at a flow rate of 0.25 mL/min. The column temperature was set at 30 °C. Mobile phases A and B were water and ACN, containing 0.1% formic acid, respectively. The gradient started at 0% B for the first 0.5 min and increased to 1% B at 9 min, followed by another increase to 95% B in 0.1 min, and held for 0.9 min to flush the column. Excipients separated by the column were detected by UV absorption at 280 nm and subjected to MS acquisition using a Q Exactive^TM^ Plus Hybrid Quadrupole-Orbitrap^TM^ Mass Spectrometer.

## 3. Results

### 3.1. Photo Degradation Pathways of DXd ADCs

We evaluated the photostability of two DXd ADC molecules, designated as ADC A and ADC B, alongside their corresponding naked antibodies, referred to antibody A and B, respectively. All four molecules were formulated in histidine buffer and subjected to cool white (CW) light exposure. The study identified four major degradation pathways of DXd ADCs following CW light exposure: formation of high-molecular-weight (HMW) species, low recovery of linker payload-conjugated peptides, post-translational modifications (PTMs), and degradation of the histidine buffer which led to formulation pH changes.

**Figure 1:**
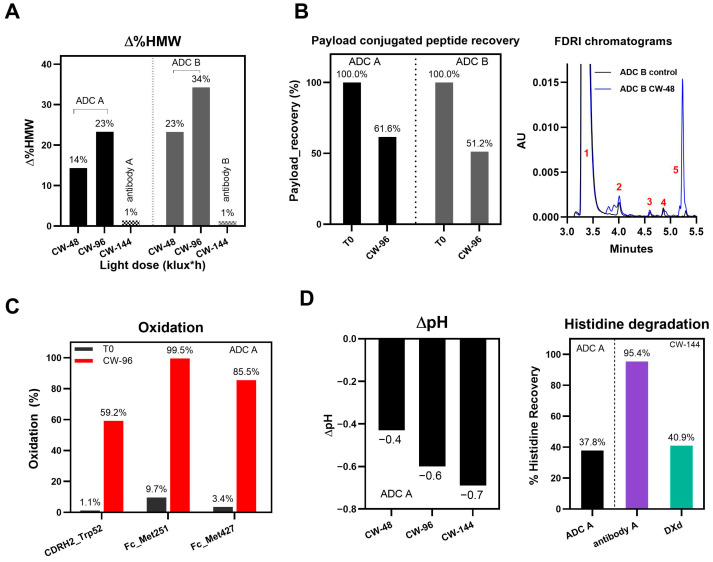
Representative degradation pathways of DXd ADCs after light exposure. (**A**) Change in %HMW (∆%HMW) of ADC A and B and their naked antibodies at various cool white (CW) light doses. (**B**) Payload-conjugated peptide recovery after CW-96 light exposure and FDRI chromatograms obtained after papain cleavage. (**C**) Oxidation levels on three residues of ADC A. (**D**) pH change (∆pH) under light exposure of CW-48, 96 and 144 klux*hours and histidine buffer degradation under CW-144 light exposure for samples in the presence of DXd ADC (black), naked antibody A (purple) and free DXd mixed with antibody A (green).

Significant increases in HMW species were observed for both ADC A and B after light exposure, with increases exceeding 20% and 30% after 96 klux*hours (CW-96) of photo exposure, respectively (Figure 1A), while only minimal increases were observed for their naked antibodies exposed to more light (144 klux*hours, CW-144). The analysis of soluble aggregates from light-exposed samples of ADC B, conducted using non-reduced (NR; treated with SDS buffer) and reduced (R; treated with both SDS buffer and DTT) microchip electrophoresis (MCE), reveals that approximately 10% of the aggregates are covalently linked (Figure S1). These covalent linkages may contribute to immunogenicity, potentially due to the formation of neo-epitopes through chemical crosslinking, as previously reported in the literature [21,22].

We also observed that, through LC-MS peptide mapping analysis, light exposure resulted in substantial loss of DXd-conjugated peptides, with only ~60% and ~50% recovery of the conjugated peptides after 96 klux*hours of exposure for ADC A and ADC B, respectively (Figure 1B). Despite this, the average DAR calculated from a SE-UPLC method based on UV absorption at 280 and 360 nm did not show meaningful change after light exposure, indicating that the linker payload barely falls off from the ADC (Table S1). However, significant amounts of DXd-conjugated peptide could not be recovered, suggesting that, while DXd remained attached to the antibody, its structure may have been altered following light exposure. To further characterize the DXd degradation products, we employed the papain deconjugation approach [23] followed by FDRI analysis coupled with LC-MS. The small-molecule drug was confirmed to be fully cleaved in the papain cleavage reaction, as demonstrated by time-course monitoring (Table S2). The FDRI chromatograms of both the control and light-exposed samples are presented in Figure 1B. The structures of the cleaved small-molecule drug (peak 1), its related impurities (peaks 2, 3 and 4) and the major photo degradant (peak 5) are shown in Table 1. These structures were confirmed through accurate mass measurement, with a precision within 10 ppm. The results revealed that the primary payload degradant in DXd ADCs exposed to light corresponds to peak 5, whose structure involved modification to the lactone/carboxylate ring of DXd. A detailed discussion of the degradant characterization will be provided in a separate manuscript.

In addition, PTMs of DXd ADCs and corresponding naked antibodies were characterized using LC-MS peptide mapping. The data showed significant oxidative modifications, histidine crosslinking, and adducts on the amino acid residues of DXd ADCs. A detailed analysis of PTMs in DXd ADCs after light exposure will be discussed in a separate manuscript. Figure 1C highlights some examples of PTMs in DXd ADC A. Methionine residues Met251 and Met427 located at the CH2-CH3 interface were oxidized to more than 85%, indicating that these residues are more susceptible to oxidation caused by DXd’s photosensitizing properties, aligning with previous observations [24]. Additionally, oxidation within the CDRs of ADC A, Trp52, was observed to reach nearly 60% after exposure to CW light of 96 klux*hours. In contrast, the changed oxidation levels on Met251, Met 427, and Trp52 in naked antibody A upon exposure to CW light of 144 klux*hours were only 8.0%, 5.0% and 1.0%, respectively.

It was observed that the pH of the histidine-buffered formulation dropped significantly upon cool white light exposure, with the extent of the pH change increasing progressively with the duration of the light exposure (Figure 1D). Further characterization revealed that the histidine buffer was significantly degraded following light exposure. The histidine recovery, calculated as the percentage change of histidine concentration of light-exposed samples compared to T0 samples, reduced to 37.6% in the ADC A sample after exposure to CW light of 144 klux*hours, compared to 95% in its naked antibody solution (Figure 1D). A similar histidine degradation level was observed for the sample with only the DXd payload in the formulation buffer, suggesting that DXd, whether conjugated or non-conjugated, is the major driver of histidine degradation (Figure 1D). Using LC-MS, several degradation products originating from the histidine buffer were detected (described in a separate manuscript mentioned above), highlighting its susceptibility to photo degradation.

In summary, four photo-induced degradation pathways of DXd ADCs were revealed. HMW formation and PTMs are common degradation pathways observed in antibodies, or other protein therapeutics under various stresses, such as thermal or photo stress. However, the levels of HMW formation and PTMs in DXd ADCs following light exposure are significantly higher than their corresponding naked antibodies. Moreover, the loss of linker payload-conjugated peptide and degradation of histidine upon light exposure represent unique degradation characteristics of DXd ADCs, attributed to the light-sensitive payload. These factors should be carefully evaluated when developing formulations, manufacturing process or risk mitigation strategies.

### 3.2. Histidine Buffer Safeguards DXd ADCs Against Light-Induced Damage

To enhance the photostability of DXd ADCs, buffer screening was performed given that the characterization of degradation pathways revealed histidine degradation occurs upon light exposure. We assessed the photostability of ADC A in citrate, phosphate, succinate, and MES buffers. A pH of 6.3 was chosen for the buffer screening of ADC A because a thermal stability study showed that ADC A exhibited optimal stability at pH 6.3 (Figure S2). Additionally, since the CPT derivative, CPT-11, was reported to exhibit a lower photo degradation rate at lower pH levels [18], we included a lower pH of 5.5 using histidine buffer for comparison.

**Figure 2:**
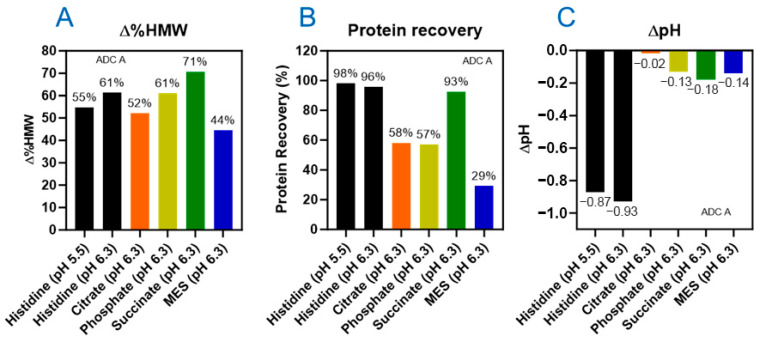
Buffer type has an impact on photostability of DXd ADCs. Varied levels of HMW increase (∆%HMW) (**A**), protein recovery (**B**), and pH change (∆pH) (**C**) observed in six buffer conditions of ADC A under light exposure of 144 klux*hours.

The SEC results showed that after 144 klux*hours of cool white light (CW-144) photo exposure, more than 40% HMW increases were observed for all formulations (Figure 2A). Among the tested buffers, citrate and MES showed slightly lower HMW increases compared to histidine buffers. However, protein recovery results, calculated from the total peak area of SEC analysis, showed that ADC lost more than 40% in citrate, phosphate, and MES buffers (Figure 2B). Consistent with the protein recovery data, samples formulated in citrate, phosphate, and MES buffers also showed increased turbidity (Figure S3). These findings suggest that citrate, phosphate, or MES buffers do not offer any advantages over histidine buffer in terms of stabilizing the ADC under light exposure. Regarding succinate formulation, light exposure resulted in increased turbidity, but higher protein recovery was observed compared to the three other buffers mentioned. However, a significantly higher level of HMW was observed compared to the histidine formulations (Figure 2B). Histidine buffer, while showing a notable pH change compared to other buffers (Figure 2C), did provide better stabilization against aggregation or protein recovery following photo exposure. Interestingly, histidine at pH 5.5 provides slightly better photostability than pH 6.3 for ADC A, as evidenced by reduced HMW formation and smaller pH change upon photo exposure.

To summarize, selecting the appropriate buffer is crucial for ADC formulation development. For DXd ADCs, incorporating photostability assessment into buffer selection studies is essential. Our data indicated that histidine buffer serves as a scavenger to protect ADC molecules from photo stress, an advantage not observed with other buffers. Despite undergoing notable pH changes, histidine buffer outperformed other buffers by providing better photostability, characterized by lower aggregation and higher protein recovery.

### 3.3. Three Excipients Significantly Enhance Photostability of DXd ADCs

**Figure 3:**
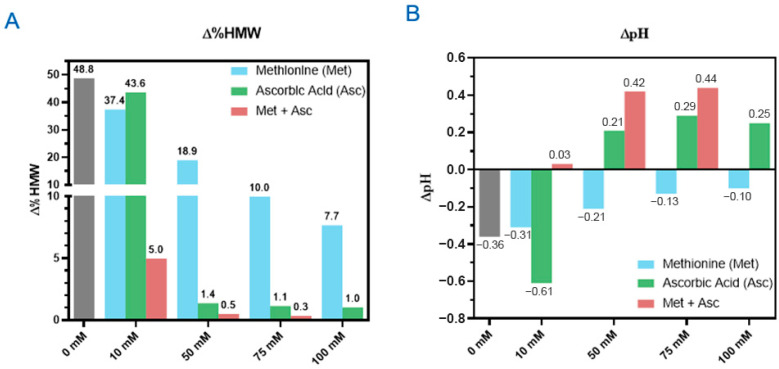
Methionine, ascorbic acid, and their combination mitigate photo degradation. Formulations using ADC B with indicated concentrations of methionine and ascorbic acid excipients were subjected to 48 klux*hours of cool white light with the changes of HMW levels (∆%HMW) (**A**) and pH (∆pH) (**B**) shown.

In the previous section, we showed that the DXd ADCs exhibited the best photostability in histidine buffer among all the buffers screened. Next, we characterized the photoprotective roles of three excipients: L-methionine, L-ascorbic acid, and sucrose. L-methionine and L-ascorbic acid are excipients often used as antioxidants in drug formulations [24]. We created a series of formulations using 25 mg/mL ADC B in histidine buffer with a concentration range of 0–100 mM for L-methionine and L-ascorbic acid, respectively. By exposing these formulations to 48 klux*hours of cool white light, we found that increasing amounts of antioxidant lowered HMW formation. Without either antioxidant, the increase in HMWs is approximately 50% (Figure 3A). At 10 mM, a slight reduction in HMW increase was observed for both antioxidants, with methionine performing slightly better than ascorbic acid. At higher concentrations, methionine was able to bring down the HMW increase close to 8% with 100 mM and maintain the pH change within 0.1 pH unit. While ascorbic acid was able to keep the HMW increase to negligible levels even at 50 mM, it had less ability to regulate the pH, as 10 mM ascorbic acid led to a pH drop of 0.6, greater than without any antioxidants, then had an increase in pH of 0.2–0.3 units for the other concentrations tested (Figure 3B). This suggests a potentially different degradation pathway compared to the pH-drop-associated degradation observed in the DXd ADC system containing no ascorbic acid. The formulations containing 50 mM or more ascorbic acid turned yellow upon light exposure, indicating oxidation of the ascorbic acid [25]. This discoloration could not only lead to difficulties in testing protein concentration and other spectrometric analysis but could also cause drug products to go out of color specification. Therefore, methionine is the preferred antioxidant compared to ascorbic acid.

We also investigated the synergistic effects of combining methionine and ascorbic acid on the photostability, using ADC B, with a concentration range of 10–75 mM for each antioxidant (100 mM methionine and 100 mM ascorbic acid not characterized) (Figure 3A). The combination of 10 mM methionine and 10 mM ascorbic acid together resulted in only a 5% increase in HMW, significantly lower than the sum of the effects observed when either excipient was used individually at the same concentration, with no change in formulation pH. However, at higher concentrations of methionine and ascorbic acid, while HMW increases were further reduced, similar pH increases and solution discoloration (yellowing) associated with higher concentrations of ascorbic acid were observed. These findings suggest that the combination of methionine and ascorbic acid has a synergistic effect in reducing HMW formation, but the pH changes and discoloration associated with higher concentrations of ascorbic acid raise concerns about its suitability as an excipient for DXd ADC formulations. Further optimization is required if the combination of ascorbic acid and methionine is to be used in the formulation of DXd ADCs.

**Figure 4:**
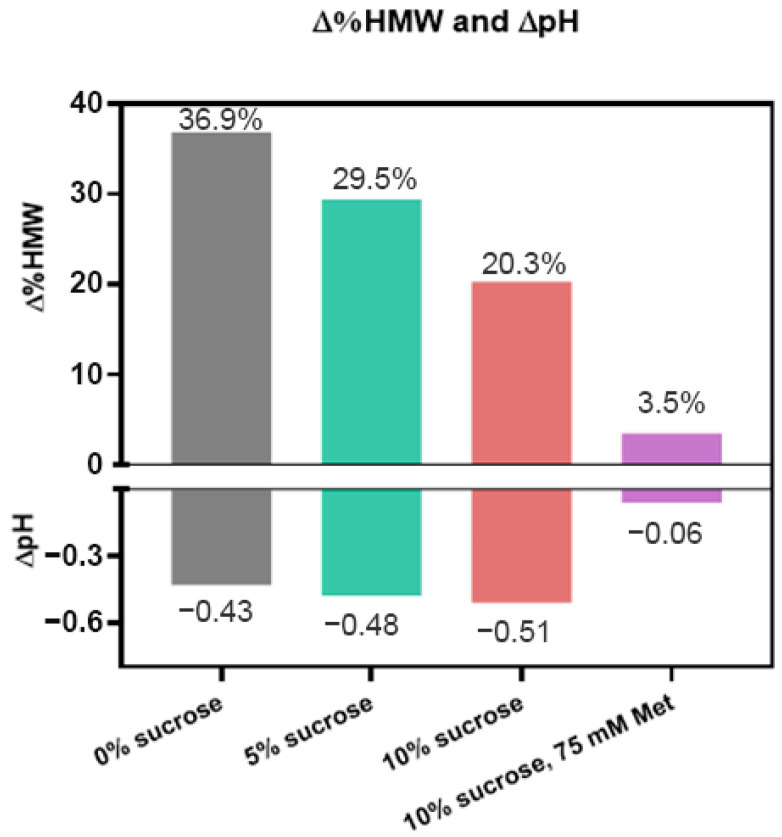
Impact on changes of HMW (∆%HMW) and pH (∆pH) of sucrose or the combination of sucrose and methionine on photostability; 25 mg/mL ADC B in a formulation of 10 mM histidine, pH 5.2, with various levels of sucrose (green and red) or a combination with methionine (purple) was subjected to 48 klux*hours of cool white light exposure.

Sucrose is a common formulation excipient used to enhance thermal stability as well as freeze/thaw stability of protein drug products. Sucrose has also been shown to have mild antioxidant properties but typically is not used in this way in pharmaceutical products [26]. We investigated the effect of sucrose on the photostability of ADC B in 10 mM histidine buffer by subjecting samples to 48 klux*hours of CW light exposure. Our results showed that without any sucrose, HMW increased by 36.9%. In contrast, it was lowered to ~30% with 5% sucrose, and ~20% with 10% sucrose added (Figure 4). However, sucrose did not improve the pH stability of the formulation, as the pH drop of all three formulations was comparable. While sucrose has been reported to have mild antioxidant activity, based on these results, the role of sucrose as a conformational stabilizer may be another reason that it could enhance photostability. Nevertheless, we conclude that the addition of sucrose at higher concentrations benefits the photostability of DXd ADCs.

We further evaluated the photostability of ADC B in the formulation containing all three excipients—10% sucrose, 75 mM methionine, and 10 mM histidine buffer—by exposing samples to 48 klux*hours of cool white light. Our results showed that the HMW increase is reduced to 3.5% with no meaningful pH change, compared with an HMW increase of 36.9% and a pH drop of 0.4 without either methionine or sucrose (Figure 4). Testing the thermal stability of this formulation, it has virtually no change in HMW at 5 °C up to 6 months, and only a 2% increase at 25 °C up to 6 months (Figure S4). Based on these results, using methionine and sucrose together significantly stabilizes the DXd ADCs when exposed to light, and also maintains their thermal stability for storage and handling.

Our findings underscore the effectiveness of excipients such as L-methionine, L-ascorbic acid, and sucrose in improving the photostability of DXd ADCs. Notably, their combined use demonstrates a substantial ability to mitigate photo degradation, thereby ensuring the preservation of ADC quality under light exposure.

### 3.4. DXd ADCs Are More Sensitive to Short-Wavelength Light

The photostability studies conducted during the excipient screening utilized cool white (CW) light emitted by a fluorescent lamp. This light predominantly covers the spectrum range of 400 to 750 nm, with minor UV-A leakage occurring between 320 and 400 nm. It is known that CPT and its derivatives have the maximum light absorption of around 370 nm [27]. To better understand whether DXd ADC molecules are specifically sensitive to certain wavelength ranges, we subjected samples to UV-A light, visible light with specific wavelengths generated using bandpass filters, and LED light (4000K). A representative LED light spectrum is shown in Supplementary Figure S5.

**Figure 5:**
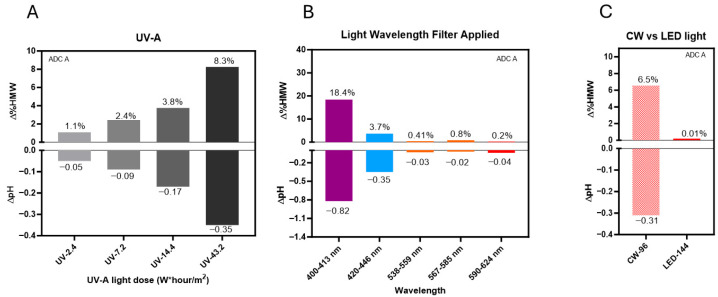
DXd ADCs are sensitive to shorter-wavelength light. (**A**) Changes of HMW species (∆%HMW) and pH (∆pH) observed in DXd ADC A with increasing doses of UV-A light exposure. (**B**) Varied HMWs and pH changes by lights with specific wavelength ranges generated using bandpass filters. (**C**) Comparison of HMWs and pH changes under CW and LED light.

Under UV-A light, photo exposure induced a dose-dependent increase in HMW formation and histidine buffer degradation (Figure 5A). Specifically, HMW levels increased from 1.1% to 8.3%, and pH dropped up to 0.35 units after exposure to 2.4 and 43.2 W*hour/m^2^ of UV-A light.

Light bandpass filters were used to study the impact of a certain narrow wavelength band on photostability of DXd ADC A. Our results showed that, when exposed to light in the 400–413 nm range, HMW levels increased by more than 18% and the pH dropped by over 0.8 units after 144 klux*hours of light exposure. On the other hand, light in the 420–446 nm range under the same light exposure led to only a 3.7% HMW increase, and a 0.35-unit pH drop. Moreover, light with wavelengths exceeding 538 nm resulted in less than 1% HMW formation and minimal pH changes, demonstrating that degradation is predominantly driven by shorter wavelengths of light (Figure 5B).

We also evaluated LED light (4000K), which contains a reduced proportion of high-energy blue light (centered at 450 nm). Our results showed that, after 144 klux*hours of LED light exposure, no noticeable HMW formation or pH change was observed (Figure 5C). By comparison, cool white light exposure at 96 klux*hours caused a 6.5% HMW increase and ~0.3 pH unit change for ADC A in the same formulation. Our data revealed that the LED light source caused minimal degradation of DXd ADCs.

Collectively, the findings indicate that DXd ADC photo instability is caused mainly by exposure to shorter-wavelength light in the UV-A and 400–450 nm range (high-energy blue light), aligned with the light absorption range of CPT-derived payloads. Furthermore, our results highlight that yellow, amber, or red light as well as LED light settings can be used as safer light sources for manufacturing, as these light sources have minimal impact on DXd ADC photostability compared with broader-spectrum white fluorescent light sources.

### 3.5. Containers Can Further Mitigate Photo Degradation of DXd ADCs

**Figure 6:**
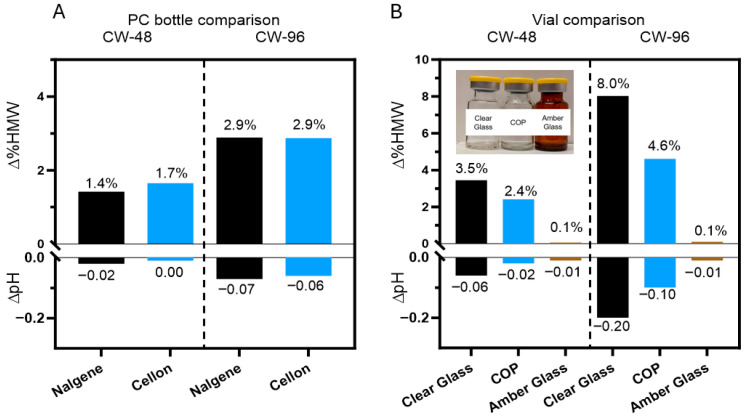
Assessment of photostability of DXd ADCs in containers for drug substances and drug products. (**A**) Comparable changes of HMWs (∆%HMW) and pH (∆pH) after photo exposure for ADC B formulation (drug substance) in both PC bottles. (**B**) Variable levels of HMW formation and pH changes observed for ADC B (drug product) in clear glass vials, COP vials, and amber glass vials.

Given the sensitivity of DXd ADCs to light exposure level and wavelength, selecting the appropriate containers for bulk drug substance and drug product production is crucial in pharmaceutical development. Container type can greatly affect the photostability of a drug, especially since different containers have varying light transmittance at certain wavelengths. Polycarbonate (PC) bottles are a common packaging material for bulk drug substances of therapeutic proteins, and they are more opaque than clear glass to some degree. However, not all PC bottles are the same, as Nalgene PC bottles have more of a blue tint than Cellon PC bottles. These two different types of PC bottles were each filled with ADC B in histidine buffer with methionine and sucrose in the formulation and exposed to 48 and 96 klux*hours of cool white light (Figure 6A). The two PC bottles had comparably low levels of HMW change at both levels of exposure, with negligible change in pH. Furthermore, both PC bottles demonstrated reduced levels of HMW formation compared to identical formulations in clear glass vials (Figure 6B). These results suggest that the selection between the two tested PC bottles will not affect the photostability of bulk drug substances.

On the drug product side, three different vials (clear glass, COP and amber glass) were tested for ADC B in histidine buffer with methionine and sucrose in the formulation by subjecting them to 48 and 96 klux*hours of cool white light exposure (Figure 6B). Clear glass vials showed the most change after light exposure to the product, as the HMW species increased by 8% after light exposure and the pH dropped by 0.2 units. The decreased UV transmittance of the COP vials had a moderate protective effect, as the HMW increased by 4.6% and the pH dropped by 0.1 unit after photo exposure. The amber glass vials demonstrated substantial mitigation of photo degradation, as the HMW and pH were both unchanged. These findings indicate that amber vials effectively shield drug products from light-induced degradation as measured by aggregation and buffer pH. As such, amber vials provide a simple yet highly effective strategy to protect DXd ADCs from light. However, as amber vials are not commonly used for biological drug products, they may require additional development and characterization, including but not limited to the challenges of implementation in the drug product manufacturing process, performing visual inspection, and managing leachables and extractables. Using amber vials is one of the options to mitigate the photo instability risk of DXd ADCs but the decision whether or not to implement this approach should be based on a comprehensive and holistic consideration.

## 4. Discussion

The photo degradation mechanisms of proteins, including therapeutic proteins, have been studied extensively [28,29,30,31]. However, the reports of characterization of ADC photostability have been limited, with only one reported study using a model ADC that employed eosin, a small-molecule surrogate, as the payload [29]. This research gap is notable given the wide use of photosensitive payloads such as camptothecin derivatives or doxorubicin in the development of ADCs [30,32,33,34]. In this study, we demonstrated that CPT-based ADCs are highly susceptible to degradation upon light exposure, which compromises the stability of the antibody, the linker payload and the excipients. Further characterization enabled the development of a robust formulation and other mitigation strategies to enhance the photostability of CPT-based ADCs during manufacturing and clinical use.

### 4.1. Photo-Induced Degradation Pathways of DXd ADCs

Our findings reveal that DXd ADCs undergo four major photo degradation pathways, including HMW aggregate formation, payload degradation, PTMs, and excipient degradation. HMW species formation [3], PTMs [4,7,12], and excipient degradation [6] have typically been observed in light-induced degradation of therapeutic antibodies. However, the extent of degradation is relatively mild compared to ADCs bearing payloads with photosensitizing features. Photosensitive payloads generally exacerbate the photo instability of ADCs by transferring energy to surrounding intra- and intermolecular structures or generating reactive species. To detect the degradation products of the linker payload upon light exposure, an enzyme-mediated deconjugation method, which cleaves DXd from ADCs, was employed as reported previously [23]. The structures of the DXd degradation products are presented in Table 1 and more details will be discussed in a separate manuscript. In addition, it is important to note that photo degradation of antibodies was observed in the presence of free DXd in solution, highlighting the need for caution during the conjugation process when free DXd is present. 

Excipient degradation upon light exposure can be particularly complex due to the coexistence of multiple excipients within the formulation system. Both our findings and the literature indicate that histidine buffer is highly susceptible to light-induced degradation. The observed decrease in pH upon light exposure is likely attributed to two key factors: the generation of acidic byproducts during the oxidation of histidine and the loss of histidine’s buffering capacity. Histidine photooxidation has been reported to directly or indirectly release protons into the solution. This can occur through mechanisms such as the decomposition of histidine hydroperoxide intermediates [35,36], as well as the formation of acidic species like trans-urocanic acid or aspartic acid [37,38]. In addition, the oxidation of histidine results in its depletion from the solution, either through the formation of crosslinked free histidine or histidine–protein adducts, further diminishing its buffering capacity [39]. Surfactants such as polysorbates (PS) are also prone to light-induced autooxidation [40,41,42]. Notably, degradation of polysorbates can be promoted by histidine buffer when residual metals exist in the system [43]. Furthermore, the level of histidine adducts upon light exposure has been reported to increase with the increasing concentration of PS20 [44]. Interestingly, in the DXd ADC system, no degradation of PS80 was observed after exposure to 144 klux*hours of CW light (details to be discussed in a separate manuscript). 

### 4.2. Stabilization of DXd ADCs Through Formulation Development

Given the inherent photosensitivity of DXd ADCs, it is crucial to develop a formulation that mitigates the four major degradation pathways to ensure drug stability during manufacturing, storage, and use. Our buffer screening studies identified histidine as the most effective buffer in protecting DXd ADCs among five buffers tested. Although our data and previous studies showed that histidine buffer undergoes degradation with pH drop upon light exposure [8], a high ADC recovery was achieved without the increase in solution turbidity. These results indicated that histidine buffer acts as an antioxidant scavenger, protecting DXd ADCs from light degradation. Employing histidine buffer to protect DXd ADCs is a viable formulation strategy, as demonstrated by our data, in addition to histidine’s established benefits as the most used formulation buffer for therapeutic antibody products.

Light exposure can impact proteins through various mechanisms, including oxygen-dependent and oxygen-independent photosensitization [45]. Mechanistically, the photo degradation of DXd ADCs can be rationalized by the excitation of DXd through light absorption, followed by photosensitization reactions, including type I and type II photosensitized oxidation reactions [46], electron transfer reactions [47], and energy transfer reactions [48]. Our study investigated the roles of formulation excipients, such as methionine and ascorbic acid, in maintaining the photostability of DXd ADCs. Water-soluble sugars such as mannitol and sucrose have demonstrated antioxidant capacity attributed to their free-radical-scavenging hydroxyl groups [49]. Our data indicates that the photo degradation level of DXd ADCs upon light exposure decreases with increasing sucrose concentration in the formulation. Although sucrose is not typically recognized as an antioxidant, possibly because its free-radical-scavenging capacity is lower compared to other antioxidants at the same molar concentration [49], it makes sense to attribute the role of sucrose in DXd ADC formulations to its dual functions as both a conformational stabilizer and antioxidant. When comparing the antioxidant roles of methionine with ascorbic acid at the same molar concentration, methionine was proven to be more effective at lower concentrations. At higher concentrations, ascorbic acid formulations resulted in lower levels of HMW formation, but the pH and color of the solution increased significantly. Although substantial knowledge exists regarding the oxidated byproducts of methionine and ascorbic acid [50,51], the degradation products that lead to pH change and discoloration in DXd ADC solutions involving histidine, sucrose, and ascorbic acid upon light exposure are complicated and beyond the scope of this report. Our results suggest that ascorbic acid is a less desirable formulation excipient for DXd ADCs due to its interference with protein concentration analysis, the more pronounced discoloration observed in ascorbic acid formulations, and decreased ability to maintain formulation pH.

Several other amino acids, such as cysteine, tryptophan, and tyrosine, have also been reported as effective excipients for photoprotective formulations [52]. In addition, exploring photoprotective excipients used in the formulation of photosensitive small-molecule drugs may offer valuable insights for the development of DXd ADC formulations. For instance, cyclodextrin has been utilized as an encapsulation carrier to enhance the photostability of small-molecule drug formulations [53]. However, its application in a DXd ADC formulation has primarily been reported to mitigate aggregation caused by agitation stress [54]. Additionally, antioxidants such as butylated hydroxytoluene (BHT) and sodium metabisulfite (SMB) have shown efficiency in the tablet formulation of simvastatin and ketoconazole, respectively, among a few other antioxidants studied [55]. We believe that multiple antioxidants may be suitable for the antioxidant role in the formulation of DXd ADCs. An effective photostabilizer should be selected based on its protective efficacy against degradation mechanisms associated with photosensitivity, its own stability when exposed to light, and the potential safety risk due to the impurities or side effects it may introduce to the formulation.

### 4.3. Other Photostabilization Strategies for DXd-ADC Development

In addition to using formulation excipients to protect DXd ADCs, another strategy involves reducing light exposure or employing safe light settings during manufacturing and handling. The maximum light absorption of CPT and its derivatives occurs at ~370 nm. Our wavelength screening study indicated that DXd ADCs are particularly sensitive to shorter wavelengths, especially UV-A and blue light, whereas wavelengths longer than 500 nm have minimal impact. The results also revealed that light settings utilizing LED lights that emit less UV-A and blue light can be safe light sources for manufacturing processes of such ADC molecules. The data confirmed that exposure to light sources outside the absorption range of CPT significantly reduces the risk of triggering photo degradation. Similar results were observed for regular antibodies, and safe lights in the manufacturing process have been proposed previously [5]. ADCs with different linker payloads may exhibit varying sensitivities to light wavelengths and could benefit from a wavelength screening study.

Reducing light exposure to DXd ADC products can also be achieved through container selection. Our study revealed that PC bottles provided better protection than clear glass vials and minimal differences in protective effects between the two types of PC bottles tested. Among the three types of drug product vials examined, our data demonstrates that amber vials can effectively prevent the damage to the ADC drug products caused by photo exposure. Thus, utilization of amber vials offers an option to mitigate photo degradation of DXd ADC drug products. However, it is important to acknowledge the limitations associated with using amber vials in biological drug products. While amber vials present a viable option, their use is not mandatory and should be carefully evaluated based on specific requirements of drug product development. Furthermore, lyophilization represents an effective strategy for mitigating the photo degradation of DXd ADC products. While this report focuses on the photo degradation of liquid DXd ADCs, a separate manuscript is in preparation to explore the role of lyophilization in enhancing the photostability of DXd ADCs.

In summary, this study highlights the critical issue of photostability in DXd ADCs, emphasizing their susceptibility to degradation upon light exposure. It identifies four major photo degradation pathways and underscores the importance of developing formulations to mitigate photo degradation. Strategies such as employing safe light sources, utilizing protective containers, and considering lyophilization are recommended. Future studies should focus on optimizing formulations and defining safe light settings for manufacturing to enhance the long-term stability of CPT-based ADCs. Moreover, it is worthwhile exploring more antioxidants and the mechanisms underlying DXd ADC stability in various formulations.

## 5. Conclusions

Photostability is a crucial aspect in the development of CPT-based ADCs, as exposure to light can lead to significant degradation. The degradation primarily involves HMW aggregate formation, linker payload degradation, PTMs on the antibody, and excipient degradation, particularly in histidine-based formulations. To mitigate these risks and improve the stability of CPT-based ADCs, several strategies can be employed, including the incorporation of antioxidants such as sucrose and methionine, the use of amber vials, the implementation of controlled light settings, and the development of lyophilized drug products. Together, these strategies provide robust solutions for improving the clinical viability of CPT-based therapeutics.

## Figures and Tables

**Table 1:** Structures of cleaved small-molecule drug, its related impurities and its photo degradation products.

FDRI Peak	Structure *
1	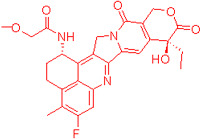
2	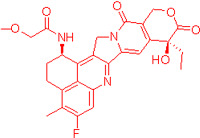
3	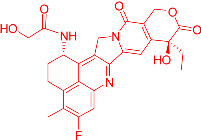
4	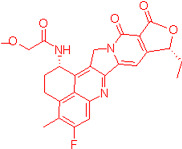
5	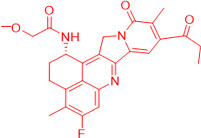

* The residue linker components after papain cleavage in peaks 1, 2, 4 and 5 were identical and are not shown in the structural representations.

## Data Availability

The original contributions presented in this study are included in the article/Supplementary Materials. Further inquiries can be directed to the corresponding authors.

## Supplementary Materials

The following supporting information can be downloaded at https://www.mdpi.com/article/10.3390/pharmaceutics17111397/s1, Figure S1: MCE analysis of ADC B with or without light exposure. (A) Non-reduced MCE profiles and (B) reduced MCE of ADC B samples before (blue) and after 48 klux*hours of cool white light (orange). The percentage of low-molecular-weight (LMW), Main, and HMW species are indicated in the table; Table S1: Average DAR of ADC B before and after light exposure; Table S2: Total amount of small molecules in the papain cleavage reactions at various time points; Figure S2: Thermal stability of ADC A in histidine buffer at various pHs; Figure S3: Impact of formulation buffer type on photostability; Figure S4: Thermal stability of ADC B in optimized formulation; Figure S5: Representative spectra of cool white and LED (4000K) light in our photostability studies.

## Abbreviations

The following abbreviations are used in this manuscript:

ADC	Antibody–drug conjugates
CPT	Camptothecin
DAR	Drug-to-antibody ratio
DXd	Deruxtecan
FDRI	Free drug-related impurities
HMW	High molecular weight
PTM	Post-translational modifications

## References

[1] Conilh L., Sadilkova L., Viricel W., Dumontet C. (2023). Payload diversification: A key step in the development of antibody-drug conjugates. J. Hematol. Oncol..

[2] Martin M., Pandiella A., Vargas-Castrillon E., Diaz-Rodriguez E., Iglesias-Hernangomez T., Martinez Cano C., Fernandez-Cuesta I., Winkow E., Perello M.F. (2024). Trastuzumab deruxtecan in breast cancer. Crit. Rev. Oncol. Hematol..

[3] Cockrell G.M., Wolfe M.S., Wolfe J.L., Schoneich C. (2015). Photoinduced aggregation of a model antibody-drug conjugate. Mol. Pharm..

[4] Shah D.D., Zhang J., Maity H., Mallela K.M.G. (2018). Effect of photo-degradation on the structure, stability, aggregation, and function of an igg1 monoclonal antibody. Int. J. Pharm..

[5] Du C., Barnett G., Borwankar A., Lewandowski A., Singh N., Ghose S., Borys M., Li Z.J. (2018). Protection of therapeutic antibodies from visible light induced degradation: Use safe light in manufacturing and storage. Eur. J. Pharm. Biopharm..

[6] Lei M., Quan C., Wang Y.J., Kao Y.-H., Schöneich C. (2018). Light-induced covalent buffer adducts to histidine in a model protein. Pharm. Res..

[7] Zhang Z., Chow S.Y., De Guzman R., Joh N.H., Joubert M.K., Richardson J., Shah B., Wikstrom M., Zhou Z.S., Wypych J. (2022). A mass spectrometric characterization of light-induced modifications in therapeutic proteins. J. Pharm. Sci..

[8] Xu C.F., Chen Y., Yi L., Brantley T., Stanley B., Sosic Z., Zang L. (2017). Discovery and characterization of histidine oxidation initiated cross-links in an igg1 monoclonal antibody. Anal. Chem..

[9] Luis L.M., Hu Y., Zamiri C., Sreedhara A. (2018). Determination of the acceptable ambient light exposure during drug product manufacturing for long-term stability of monoclonal antibodies. PDA J. Pharm. Sci. Technol..

[10] Sharma B. (2007). Immunogenicity of therapeutic proteins. Part 3: Impact of manufacturing changes. Biotechnol. Adv..

[11] Perez Medina Martinez V., Robles M.C., Juarez-Bayardo L.C., Espinosa-de la Garza C.E., Meneses A., Perez N.O. (2022). Photodegradation of rituximab and critical evaluation of its sensibility to electromagnetic radiation. AAPS PharmSciTech.

[12] Lam X.M., Yang J.Y., Cleland J.L. (1997). Antioxidants for prevention of methionine oxidation in recombinant monoclonal antibody her2. J. Pharm. Sci..

[13] Wang W., Vlasak J., Li Y., Pristatsky P., Fang Y., Pittman T., Roman J., Wang Y., Prueksaritanont T., Ionescu R. (2011). Impact of methionine oxidation in human igg1 fc on serum half-life of monoclonal antibodies. Mol. Immunol..

[14] Bertolotti-Ciarlet A., Wang W., Lownes R., Pristatsky P., Fang Y., McKelvey T., Li Y., Li Y., Drummond J., Prueksaritanont T. (2009). Impact of methionine oxidation on the binding of human igg1 to fc rn and fc gamma receptors. Mol. Immunol..

[15] Pan H., Chen K., Chu L., Kinderman F., Apostol I., Huang G. (2009). Methionine oxidation in human igg2 fc decreases binding affinities to protein a and fcrn. Protein Sci..

[16] Walton W.J., Zhang S.J., Wilson J.J., Harvey B.N., Clemens M., Gu Y. (2025). Impact of monoclonal antibody aggregates on effector function characterization. Antibodies.

[17] Lown J.W., Hsiao-Hsiung C. (1980). Studies on the effects of the antitumor agent camptothecin and derivatives on deoxyribonucleic acid: Mechanism of the scission of deoxyribonucleic acid by photoactivated camptothecin. Biochem. Pharmacol..

[18] Dodds H.M., Craik D.J., Rivory L.P. (1997). Photodegradation of irinotecan (cpt-11) in aqueous solutions: Identification of fluorescent products and influence of solution composition. J. Pharm. Sci..

[19] Mahalingaiah P.K., Ciurlionis R., Durbin K.R., Yeager R.L., Philip B.K., Bawa B., Mantena S.R., Enright B.P., Liguori M.J., Van Vleet T.R. (2019). Potential mechanisms of target-independent uptake and toxicity of antibody-drug conjugates. Pharmacol. Ther..

[20] Beck A., Goetsch L., Dumontet C., Corvaia N. (2017). Strategies and challenges for the next generation of antibody-drug conjugates. Nat. Rev. Drug Discov..

[21] Paul R., Graff-Meyer A., Stahlberg H., Lauer M.E., Rufer A.C., Beck H., Briguet A., Schnaible V., Buckel T., Boeckle S. (2012). Structure and function of purified monoclonal antibody dimers induced by different stress conditions. Pharm. Res..

[22] Bessa J., Boeckle S., Beck H., Buckel T., Schlicht S., Ebeling M., Kiialainen A., Koulov A., Boll B., Weiser T. (2015). The immunogenicity of antibody aggregates in a novel transgenic mouse model. Pharm. Res..

[23] Li Y., Gu C., Gruenhagen J., Yehl P., Chetwyn N.P., Medley C.D. (2016). An enzymatic deconjugation method for the analysis of small molecule active drugs on antibody-drug conjugates. MAbs.

[24] Liu D., Ren D., Huang H., Dankberg J., Rosenfeld R., Cocco M.J., Li L., Brems D.N., Remmele R.L. (2008). Structure and stability changes of human igg1 fc as a consequence of methionine oxidation. Biochemistry.

[25] Du J., Cullen J.J., Buettner G.R. (2012). Ascorbic acid: Chemistry, biology and the treatment of cancer. Biochim. Biophys. Acta.

[26] Ji J.A., Zhang B., Cheng W., Wang Y.J. (2009). Methionine, tryptophan, and histidine oxidation in a model protein, pth: Mechanisms and stabilization. J. Pharm. Sci..

[27] Ziomkowska B. (2006). Deactivation rate of camptothecin determined by factor analysis of steady-state fluorescence and absorption spectra. Opt. Appicata.

[28] Qi P., Volkin D.B., Zhao H., Nedved M.L., Hughes R., Bass R., Yi S.C., Panek M.E., Wang D., Dalmonte P. (2009). Characterization of the photodegradation of a human igg1 monoclonal antibody formulated as a high-concentration liquid dosage form. J. Pharm. Sci..

[29] Sreedhara A., Yin J., Joyce M., Lau K., Wecksler A.T., Deperalta G., Yi L., John Wang Y., Kabakoff B., Kishore R.S. (2016). Effect of ambient light on igg1 monoclonal antibodies during drug product processing and development. Eur. J. Pharm. Biopharm..

[30] Hernandez-Jimenez J., Salmeron-Garcia A., Cabeza J., Velez C., Capitan-Vallvey L.F., Navas N. (2016). The effects of light-accelerated degradation on the aggregation of marketed therapeutic monoclonal antibodies evaluated by size-exclusion chromatography with diode array detection. J. Pharm. Sci..

[31] Schoneich C. (2020). Photo-degradation of therapeutic proteins: Mechanistic aspects. Pharm. Res..

[32] Gęgotek A., Skrzydlewska E., Litwack G. (2023). Chapter nine—Ascorbic acid as antioxidant. Vitamins and Hormones.

[33] Peng M., Liu Y., Zhang H., Cui Y., Zhai G., Chen C. (2010). Photostability study of doxorubicin aqueous solution enhanced by inclusion interaction between doxorubicin and hydroxypropyl-β-cyclodextrin. Chin. J. Chem..

[34] Syed Y.Y. (2020). Sacituzumab govitecan: First approval. Drugs.

[35] Matsukawa R., Yamane M., Kanai M. (2023). Histidine photooxygenation chemistry: Mechanistic evidence and elucidation. Chem. Rec..

[36] Agon V.V., Bubb W.A., Wright A., Hawkins C.L., Davies M.J. (2006). Sensitizer-mediated photooxidation of histidine residues: Evidence for the formation of reactive side-chain peroxides. Free Radic. Biol. Med..

[37] Wang C., Yamniuk A., Dai J., Chen S., Stetsko P., Ditto N., Zhang Y. (2015). Investigation of a degradant in a biologics formulation buffer containing l-histidine. Pharm. Res..

[38] Miyahara Y., Shintani K., Hayashihara-Kakuhou K., Zukawa T., Morita Y., Nakazawa T., Yoshida T., Ohkubo T., Uchiyama S. (2020). Effect of uvc irradiation on the oxidation of histidine in monoclonal antibodies. Sci. Rep..

[39] Liu M., Zhang Z., Cheetham J., Ren D., Zhou Z.S. (2014). Discovery and characterization of a photo-oxidative histidine-histidine cross-link in igg1 antibody utilizing ^18^O-labeling and mass spectrometry. Anal. Chem..

[40] Weber J., Buske J., Mader K., Garidel P., Diederichs T. (2023). Oxidation of polysorbates—An underestimated degradation pathway?. Int. J. Pharm. X.

[41] Kerwin B.A. (2008). Polysorbates 20 and 80 used in the formulation of protein biotherapeutics: Structure and degradation pathways. J. Pharm. Sci..

[42] Donbrow M., Azaz E., Pillersdorf A. (1978). Autoxidation of polysorbates. J. Pharm. Sci..

[43] Gopalrathnam G., Sharma A.N., Dodd S.W., Huang L. (2018). Impact of stainless steel exposure on the oxidation of polysorbate 80 in histidine placebo and active monoclonal antibody formulation. PDA J. Pharm. Sci. Technol..

[44] Lei M., Quan C., Wang J.Y., Kao Y.H., Schoneich C. (2021). Light-induced histidine adducts to an igg1 molecule via oxidized histidine residue and the potential impact of polysorbate-20 concentration. Pharm. Res..

[45] Baptista M.S., Cadet J., Greer A., Thomas A.H. (2021). Photosensitization reactions of biomolecules: Definition, targets and mechanisms. Photochem. Photobiol..

[46] Baptista M.S., Cadet J., Di Mascio P., Ghogare A.A., Greer A., Hamblin M.R., Lorente C., Nunez S.C., Ribeiro M.S., Thomas A.H. (2017). Type i and type ii photosensitized oxidation reactions: Guidelines and mechanistic pathways. Photochem. Photobiol..

[47] Escudero D. (2016). Revising intramolecular photoinduced electron transfer (pet) from first-principles. Acc. Chem. Res..

[48] Liu J., Wei Y., Ma L., Jin X., Sun S., Liu M., Du J., Xu X., Tian H., Ma X. (2024). Photo-induced energy transfer polymerization. ChemRxiv.

[49] Van den Ende W., Valluru R. (2009). Sucrose, sucrosyl oligosaccharides, and oxidative stress: Scavenging and salvaging?. J. Exp. Bot..

[50] Kim G., Weiss S.J., Levine R.L. (2014). Methionine oxidation and reduction in proteins. Biochim. Biophys. Acta.

[51] Njus D., Kelley P.M., Tu Y.J., Schlegel H.B. (2020). Ascorbic acid: The chemistry underlying its antioxidant properties. Free Radic. Biol. Med..

[52] Kawabata K., Kanoh M., Okazaki M., Maeda R., Mori S., Akimoto S., Inagaki M., Nishi H. (2020). Photoprotective effects of selected amino acids on naproxen photodegradation in aqueous media. Pharmaceuticals.

[53] Detoni C.B., Souto G.D., da Silva A.L., Pohlmann A.R., Guterres S.S. (2012). Photostability and skin penetration of different e-resveratrol-loaded supramolecular structures. Photochem. Photobiol..

[54] Johann F., Woll S., Gieseler H. (2024). Evaluating the potential of cyclodextrins in reducing aggregation of antibody-drug conjugates with different payloads. J. Pharm. Sci..

[55] Celestino M.T., Magalhães U.d.O., Fraga A.G.M., Carmo F.A.d., Lione V., Castro H.C., Sousa V.P.d., Rodrigues C.R., Cabral L.M. (2012). Rational use of antioxidants in solid oral pharmaceutical preparations. Braz. J. Pharm. Sci..

