# Supplementary material for: Insights into Photo Degradation and Stabilization Strategies of Antibody–Drug Conjugates with Camptothecin Payloads
Source: Pharmaceutics. 2025 Oct 28;17(11):1397. doi: 10.3390/pharmaceutics17111397 (PMC12655814; doi:10.3390/pharmaceutics17111397)
Supplement: Supplementary file 1 [file pharmaceutics-17-01397-s001.zip › pharmaceutics-3885909-supplementary.pdf]

pharmaceutics

MDPI

Supporting Information

Insights into Photo Degradation and Stabilization Strategies of Antibody–Drug Conjugates with Camptothecin Payloads

Shukun Luo [1,†], Joshua Bulos [1,†], Ricky Uroza [1], Yimeng Zhao [2], Xiao Pan [2], Yue Su [2], Haibo Qiu [2], Babatunde Olagunju [1,3], Wenhua Wang [1,*], Dingjiang Liu [1,*] and Mohammed Shameem [1]

[1] Formulation Development, Regeneron Pharmaceuticals, Inc., 777 Old Saw Mill River Road, Tarrytown, NY 10591, USA; shukun.luo@regeneron.com (S.L.); joshua.bulos@regeneron.com (J.B.); ricky.uroza@regeneron.com (R.U.); femtuns88@gmail.com (B.O.); mohammed.shameem@regeneron.com (M.S.)
[2] Analytical Chemistry Group, Regeneron Pharmaceuticals, Inc., 777 Old Saw Mill River Road, Tarrytown, NY 10591, USA; yimeng.zhao@regeneron.com (Y.Z.); xiao.pan@regeneron.com (X.P.); yue.su@regeneron.com (Y.S.); haibo.qiu@regeneron.com (H.Q.)
[3] Department Of Chemistry, State University of New York College of Environmental Science and Forestry, Syracuse, NY 13210, USA
* Correspondence: wenhua.wang@regeneron.com (W.W.); dingjiang.liu@regeneron.com (D.L.)
† Authors contributed equally to this work.

Results

Photo degradation pathways of DXd ADCs

NR-MCE and R-MCE analysis were performed on T0 control and light-exposed samples of ADC B to investigate the nature of HMW species. For NR-MCE, samples were treated with sodium dodecyl sulfate (SDS), an anionic detergent that disrupts non-covalent association while preserving the disulfide bonds within proteins. Meanwhile, R-MCE samples were treated with both SDS and dithiothreitol (DTT), where DTT acts as a reducing agent to break disulfide bonds. The analysis revealed approximately 10% of HMW species in the light-exposed samples in both NR-MCE and R-MCE. These HMW species are likely covalently linked, which could present a potential immunogenicity risk.

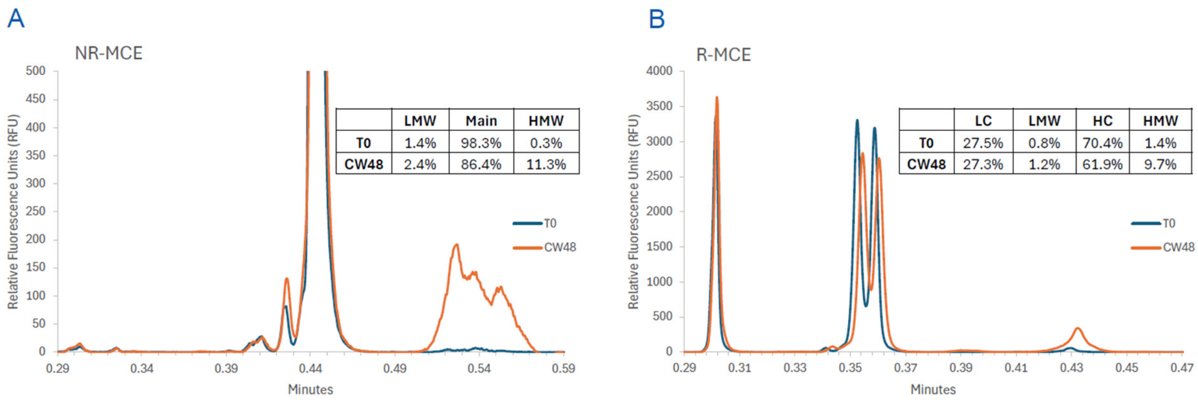

Figure S1. MCE analysis of ADC B with or without light exposure. (**A**) Non-reduced MCE profiles and (**B**) reduced MCE of ADC B samples before (blue) and after 48 klux*hours of cool white light (orange). The percentage of low-molecular-weight (LMW), Main, and HMW species are indicated in the table.

Regeneron - Internal

The average drug-to-antibody ratio (DAR) of ADC B, measured by SE-UPLC before and after light exposure, is presented in Table S1. The data indicated no meaningful change in the average DAR following light exposure.

Table S1. Average DAR of ADC B before and after light exposure.

ADC B	Control	96 klux*hours cool white light exposure
DAR	3.85	3.80

Papain cleavage studies were conducted with reactions monitored at 10, 30, and 60 minutes. The total amounts of the cleaved small-molecule drug and its derivatives detected by FDRI are summarized in Table S2. The data demonstrated that full cleavage was completed within 10 minutes.

Table S2. Total amount of small molecules in the papain cleavage reactions at various time points.

Reaction time	10 minutes	30 minutes	60 minutes
Total amount of small molecules (ug/mL) by FDRI	230.5	224.3	240.4

Histidine buffer safeguards DXd ADCs against light-induced damage

Samples of ADC A formulated with 10 mM histidine at three different pH levels and 5% sucrose were subject to thermal stress at 40 °C for 14 days. The HMW species were analyzed using SEC-UPLC, as shown in Figure S2. The results indicated that ADC A showed optimal thermal stability at pH 6.3.

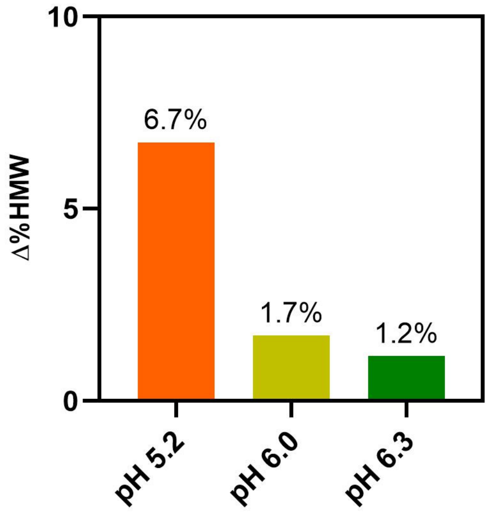

Figure S2. Thermal stability of ADC A in histidine buffer at various pHs.

Figure S3 illustrates the visual appearance of ADC A formulated in various buffer types upon light exposure. For comparison, samples prepared in histidine formulations at pH 5.5 and pH 6.3 prior to light exposure were also presented. Notable differences in turbidity and precipitation levels were observed across citrate, phosphate, succinate, and MES buffers.

Regeneron - Internal

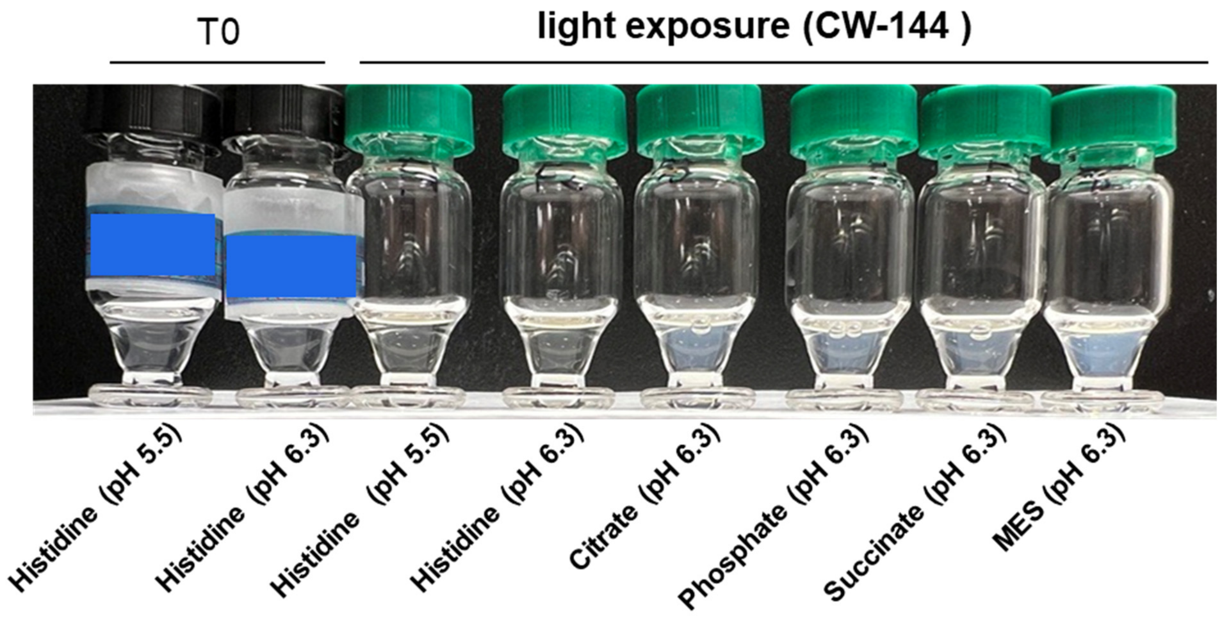

Figure S3. Impact of formulation buffer type on photostability.

Three excipients significantly enhance photostability of DXd ADCs

The thermal stability of ADC B, formulated in 10 mM histidine, 75 mM methionine, and 10% sucrose, was assessed in liquid form at 5 °C and 25 °C for up to 6 months. The HMW species were analyzed using SE-UPLC, as shown in Figure S4. The results showed no meaningful change at 5 °C for up to 6 months and an approximately 0.5% increase in HMW species at 25 °C after one month. These findings highlight that the optimized formulation, developed through buffer and excipient screening based on photostability, also demonstrates good thermal stability.

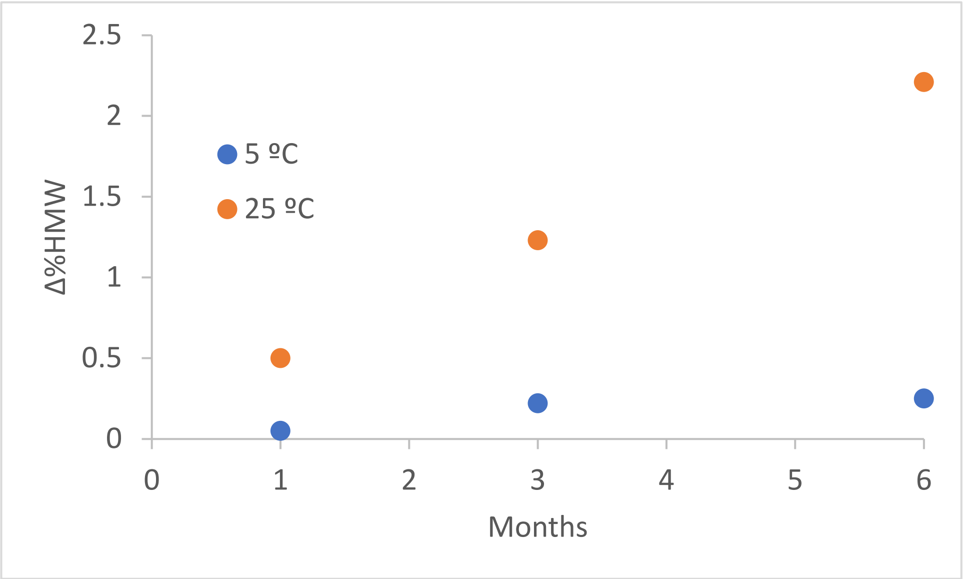

Figure S4. Thermal stability of ADC B in optimized formulation.

DXd ADCs are more sensitive to short-wavelength light

The wavelength ranges of UV-A and cool white, fluorescent light, as utilized in the Bahnson photo stability chamber, along with the LED light spectrum representing our

Regeneron - Internal

self-assembled photo chamber are depicted in Figure S5. Additionally, the high-energy blue light was also indicated.

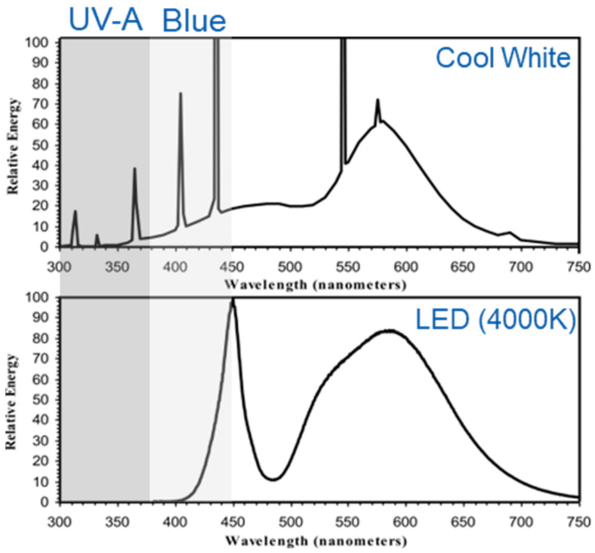

Figure S5. Representative spectra of cool white and LED (4000K) light in our photostability studies. Dark grey area represents the UV-A spectra, and light grey represents the spectra extending up to blue light.

Regeneron - Internal
